# Oral CT Image Processing Based on Oral CT Image Filtering Algorithm

**DOI:** 10.1155/2022/6041872

**Published:** 2022-09-19

**Authors:** Jiyong Yang, Cheng Wang, Jun Xiang, Binbin Hu, Kun Yang, Na Li

**Affiliations:** ^1^Department of Stomatology, Renhe Hospital of China Three Gorges University, Yichang 443000, Hubei Province, China; ^2^Department of Medical Imaging Science, Renhe Hospital of China Three Gorges University, Yichang 443000, Hubei Province, China

## Abstract

The application of computer vision technology in the medical field provides more accurate technical support for oral disease detection. The research proposes the CT image denoising algorithm based on wavelet and bilateral filtering. Through the study of the imaging principle of CT image and the CT image acquisition scene, the CT image data is filtered by wavelet and bilateral filtering algorithm, and the algorithm is proposed from the peak signal-to-noise ratio, structural similarity, and the effective detection of three-dimensional image construction of the image. The test results show that the proposed algorithm has excellent performance in the aspects of peak signal-to-noise ratio, structural similarity, and error of mean square. When the proportions of Gaussian noise are 10%, 20%, 30%, 40%, and 50%, the MSE error values of the proposed algorithm are 0.002, 0.004, 0.006, and 0.007, respectively, and the performance is the best in the comparison of multiple algorithms. The contents of the research provide an important theoretical reference for the treatment of clinical oral diseases.

## 1. Introduction

The problems of oral health have always been highly concerned by modern people. Oral diseases not only affect people's physical and mental health, but also affect their work and life. With the continuous development of modern computer science and technology, the application of computer vision processing technology in the field of stomatology has provided important technical support for medical workers [1, 2]. Computer vision technology, based on artificial intelligence technology and computer graphics processing technology, solves the problem of lack of medical working experience through intelligence and automation. CT technology, also known as microscopic scanning technology, can build a three-dimensional visualization model through scanning patients in the medical field, helping medical workers to more accurately confirm the patient's condition [3, 4]. However, traditional CT has a lot of noise problems. The application of computer vision technology in CT images will make up for the problems of traditional CT images with much noise and poor quality, and greatly improve the diagnostic effect of medical workers [5]. In the future, computer artificial intelligence technology will be widely used in the modern medical field, and intelligent oral technology will become the focus of the development of the oral field.

## 2. Construction of CT Image Model Based on Oral CT Image Filtering Algorithm

### 2.1. Construction of CT Image Denoising Model

The CT imaging technology is an important industry in the development of modern medicine. The three-dimensional view of the human body is constructed by scanning the parts of the human body with X-rays. The grayscale of CT imaging will reflect the absorption intensity of X-rays by various organs of the patient, thereby helping medical workers accurately judge the condition [6–8]. Due to the imaging principle of CT image and the complex environmental factors of collecting the data of patients, it will lead to the problem of uneven grayscale or grayscale difference in CT imaging, which cannot help medical workers more accurately judge the symptoms of patients.

In the process of oral CT scanning, there is a lot of noise and unclear images in the actual obtained CT images. In the process of oral treatment, the noise problem of CT image has brought many impacts on the examination of oral diseases by doctors, so it is necessary to denoise CT scanning images [9, 10]. The filtering algorithms used in different CT images are different in image denoising. If the CT image filtering algorithm is arbitrarily selected for denoising, the effect of the final image denoising will not be satisfactory [11]. Therefore, in the process of denoising the oral CT image, it is necessary to identify the noise type of the image. There are various noises in the images obtained from CT scanning. Different noises require different denoising methods. According to the principle of image degradation, the noise types are mainly additive noise and multiplicative noise. The expression of additive noise is shown in the following formula:

(1) $$\begin{array}{c} g\left(i,j\right)=x\left(i,j\right)+n\left(i,j\right)\text{.} \end{array}$$

In formula (1), *g*(*i*, *j*) represents the actual output image, *n*(*i*, *j*) represents the noise image, and *x*(*i*, *j*) represents the ideal image. The expression of multiplicative noise is shown in the following formula:

(2) $$\begin{array}{c} g\left(i,j\right)=x\left(i,j\right)\left[1+n\left(i,j\right)\right]\text{.} \end{array}$$

In the analysis of image denoising types, the noise is divided into two types from the perspective of probability and statistics. One is impulse noise, and the other one is Gaussian noise. Among them, Gaussian noise needs to conform to the Gaussian distribution law. For this kind of noise, it is taken as a variable in the processing, and the representation variable is used to obtain the probability density function in the image as shown in the following formula:

(3) $$\begin{array}{c} p\left(z\right)=\frac{1}{\sqrt{2\pi \sigma }}e^{-\left(z-\mu \right)/\left(2\delta^{2}\right)}\text{.} \end{array}$$

In formula (3), *σ* is the standard deviation, *μ* is the mean value, and *δ*^2^ is the variance. At the same time, the probability density of impulse noise is shown in formula (5).

(4) $$\begin{array}{c} p\left(z\right)\left\{\begin{array}{c} p_{a};\;z=a\\ p_{b};\;z=b\\ 0;\;z=else \end{array}\right.\text{.} \end{array}$$

In formula (4), if *b* > *a* exists, it indicates that there is a bright spot with grayscale *b* in the image, and *a* represents a dark spot in the image. At the same time, if the value of *p*_*a*_ or *p*_*b*_ is 0, the image can be called an impulse noise image. If the mean values of *p*_*a*_ and *p*_*b*_ are both not 0, and the two values are similar, the noise in the image will be distributed in the form of random noise and distributed in the salt powder particles and pepper. Therefore, impulse noise is also called salt pepper noise [12–14].

**Figure 1:**
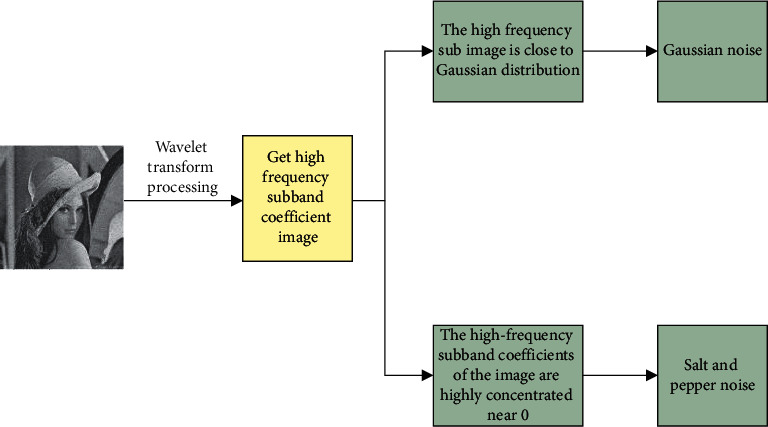
Judgment of CT noise image.

Considering that there is a lot of noise in oral images, a suitable filtering algorithm should be chosen for the image noise processing. Only in this way can the image be effectively denoised. Therefore, in the process of image denoising, it is necessary to reasonably judge the type of image noise. In the division of CT image noise, image domain noise and projection domain noise are included. The projection domain noise belongs to the multiplicative noise that depends on signal strength. For the image domain noise, it is mainly similar to Gaussian white noise. The judgment of CT noise image is shown in Figure 1.

### 2.2. Construction of CT Image Processing Model Based on Wavelet Transformation and Bilateral Filtering

Originally applied in the field of signal and mathematical processing, wavelet is a filter that locates a signal at different levels or emission thresholds. With the development of computer vision technology, wavelet has been widely applied in the field of multiscale directed filtering. To study the CT image denoising algorithm based on wavelet and bilateral filtering, firstly, wavelet transformation is required, mainly referring to the local transformation in the time domain and emission domain, which is conducive to better analysis and extraction of image feature data [15, 16]. In the process of wavelet transformation, the image will be decomposed in the form of wavelet, and the number of layers of wavelet processing will be set, which is more conducive to the control of the amount of image calculation. The corresponding one-dimensional discrete wavelet function will be set as *ψ*_*j*,*k*_(*x*) and *ψ*_*j*,*l*_(*y*), so the two-dimensional discrete wavelet expression is obtained as shown in the following formula:

(5) $$\begin{array}{c} \left\{\begin{array}{c} \varphi_{A}\left(x,y\right)=\varphi_{j,k}\left(x\right)\varphi_{j,l}\left(y\right)\\ \psi_{H}\left(x,y\right)=\varphi_{j,k}\left(x\right)\psi_{j,l}\left(y\right)\\ \psi_{V}\left(x,y\right)=\psi_{j,k}\left(x\right)\varphi_{j,l}\left(y\right)\\ \psi_{D}\left(x,y\right)=\psi_{j,k}\left(x\right)\psi_{j,l}\left(y\right) \end{array}\right.\text{.} \end{array}$$

**Figure 2:**
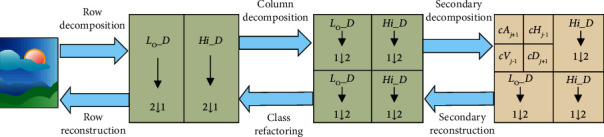
Two-dimensional wavelet discrete decomposition.

In formula (5), *φ*_*A*_(*x*, *y*) represents two-dimensional discrete scale function, *ψ*_*H*_(*x*, *y*) represents the horizontal corresponding two-dimensional discrete wavelet function, *ψ*_*V*_(*x*, *y*) represents the vertical corresponding two-dimensional discrete wavelet function, and *ψ*_*D*_(*x*, *y*) represents diagonal detail corresponding wavelet function. In image wavelet transformation, two-dimensional small functions can be transformed by two one-dimensional wavelet functions. In the wavelet transformation processing, the discrete wavelet transformation processing should be carried out for each row of the matrix first, and then discrete wavelet transform processing should be carried out for each column of the image, so that the two-dimensional wavelet transformation processing can be completed. The specific decomposition process is shown in Figure 2.

In Figure 2, the results of two-dimensional discrete decomposition include a horizontal detail component c*H*, approximate component *cA*, diagonal component *cD*, and vertical detail component *cV*, in which 2 ↓ 1 is line reduction, and the number of rows is even. The results of two-dimensional wavelet transformation are used to reconstruct images at different scales to realize two-dimensional discrete wavelet inverse transformation. At the same time, the wavelet transformation in the lower layer of the image is completed by the low-frequency subimage generated in the upper layer. By repeating it in sequence, the decomposition of multi-layer two-dimensional discrete wavelet of the image can be realized [17, 18]. In addition, every time the image wavelet transformation is processed, the vertical and horizontal directions of the image will be sampled at corresponding intervals. Therefore, the image will be decomposed into four foreground band subimages in the transformation process.

The wavelet denoising is carried out after the wavelet transformation. The coefficients of wavelet decomposition will be further processed, and the wavelet coefficients will be reconstructed at the same time, so as to obtain the suppression and processing of the image noise. Among them, the threshold denoising is the most common form of wavelet denoising, which needs to consider the selection of threshold and threshold processing function. Donoho will process the threshold and divide it into the soft and hard thresholds [19].

According to the principle of wavelet processing, the original image obtained by CT scanning is set as ω, and *ω*_*λ*_ is obtained by applying the wavelet coefficient to the threshold, with *λ* representing the threshold limit. The next step of soft threshold processing is to set the wavelet coefficient within the threshold to 0. If the wavelet coefficient is greater than the set threshold, further processing is required as shown in formula (2).

(6) $$\begin{array}{c} \omega_{\lambda }\left\{\begin{array}{c} \sin\;gn\left(\omega \right)\left(\left|\omega \right|-\lambda \right)\left|\omega \right|\geq \lambda ,\\ 0\left|\omega \right|\leq \lambda . \end{array}\right. \end{array}$$

In the processing of wavelet hard threshold, the wavelet coefficient that is less than the threshold authority is also set to 0, while for the threshold that is greater than the wavelet coefficient, there is no need to process it. Then the expression is shown in formula (3).

(7) $$\begin{array}{c} \omega_{\lambda }\left\{\begin{array}{c} \omega \left|\omega \right|\geq \lambda ,\\ 0\left|\omega \right|<\lambda . \end{array}\right. \end{array}$$

In wavelet processing, the threshold set in the hard threshold is not completely continuous. There is a phenomenon of oscillation in the process of function processing, which leads to the problem of distortion in the image processing. But the soft threshold function coefficient is connected smoothly. However, due to the coefficient shrinkage processing, there is a blurring problem in the processing of the image's edge. Therefore, it is necessary to improve the defects of the traditional threshold function, such as using the soft and hard thresholds compromise function and adding an adjustment coefficient *a*(0 ≤ *a* ≤ 1) to the threshold function. After adding the adjustment coefficient, the applied threshold will be between the soft and hard thresholds of the wavelet coefficient *ω*_*λ*_ , which also makes the wavelet coefficient closer to the real wavelet coefficient. The expression after processing is shown in the following formula:

(8) $$\begin{array}{c} \omega_{\lambda }\left\{\begin{array}{c} \sin\;g\left(\omega \right)\left(\left|\omega \right|-a\lambda \right)\left|\omega \right|\geq \lambda ,\\ 0\left|\omega \right|<\lambda . \end{array}\right. \end{array}$$

In formula (8), when *a* = 1 (0 ≤ *a* ≤ 1), it is a soft threshold function; if *a*= = 0, it is a hard threshold function. In the wavelet image denoising, for the smaller wavelet coefficient, the overall proportion of wavelet coefficients is not high. In the comparison of the above wavelet function thresholds, the relatively small coefficients are set to 0, which will have a loss effect on the signal. In this regard, the soft and hard threshold compromise function will be optimized, and the improved expression is shown in the following formula:

(9) $$\begin{array}{c} \omega_{\lambda }\left\{\begin{array}{c} \sin\;g\left(\omega \right)\left(\left|\omega \right|-\left(1-a\right)\lambda \right)\left|\omega \right|\geq \lambda \\ a\lambda \left|\omega \right|<\lambda \end{array}\right.\text{.} \end{array}$$

In formula (9), when *a*=0 ( 0 ≤ *a* ≤ 1 ), the threshold function will become a soft threshold function. Multiplying the smaller wavelet coefficient with a constant will effectively retain part of the useful signals, retain the details of image processing, and effectively reduce the mean square error generated by the signal reconstruction. The expression of fixed threshold is shown in the following formula:

(10) $$\begin{array}{c} \left\{\begin{array}{c} T=\sigma \sqrt{2\;\log\left(N\right)}\\ \sigma =me\;di\;an\left(y\right)/0.674 \end{array}\right.\text{.} \end{array}$$

In formula (10), *N* represents the signal scale and *σ* represents the variance of noise. In threshold denoising, Bayes threshold estimation belongs to a relatively effective form of threshold denoising, and meets the requirements of Gaussian distribution. The expression of Bayes threshold estimation is shown in the following formula:

(11) $$\begin{array}{c} \left\{\begin{array}{c} T=\min\left(J\right)\\ J=\frac{1}{N}{\sum \left(w-w_{\sigma }\right)}^{2}/\left(\frac{N_{0}}{N}\right) \end{array}\right.\text{.} \end{array}$$

In formula (11), *N* represents the number of wavelet coefficients, *N*_0_ represents the number of 0 wavelet coefficient set after the wavelet shrinkage, *w* is the coefficient before the shrinkage, and *w*_*σ*_ is the coefficient after the shrinkage.

Bilateral filter belongs to a noniterative and nonlinear filter, which is a variant of Gaussian filter. It is widely used in the field of image denoising at present, and ensures that the image retains more details. In the process of image processing, it depends on the weighted combination of neighborhood pixel values. Its expression is shown in the following formula:

(12) $$\begin{array}{c} g\left(x,y\right)=\frac{\sum {}_{k,j}f\left(k,l\right)\omega \left(i,j,k,l\right)}{\sum {}_{k,j}\omega \left(i,j,k,l\right)}\text{.} \end{array}$$

In formula (12), *ω*(*i*, *j*, *k*, *l*) represents the weight coefficient and *f*(*k*, *l*) represents the adjacent pixel function. The weight coefficient is determined by the product of the value domain kernel *r*(*i*, *j*, *k*, *l*) in the definition domain kernel (*i*, *j*, *k*, *l*), and the specific expression is shown in the following formula:

(13) $$\begin{array}{c} \left\{\begin{array}{c} d\left(i,j,k,l\right)=\exp\;\left(-\frac{{\left(i-k\right)}^{2}+{\left(j-l\right)}^{2}}{2\sigma_{d}^{2}}\right)\\ r\left(i,j,k,l\right)=\exp\;\left(-\frac{{\left\|f\left(i-j\right)-f\left(k-l\right)\right\|}^{2}}{2\sigma_{r}^{2}}\right)\\ \omega \left(i,j,k,l\right)=\exp\;\left(-\frac{{\left(i-k\right)}^{2}+{\left(j-l\right)}^{2}}{2\sigma_{d}^{2}}-\frac{{\left\|f\left(i-j\right)-f\left(k-l\right)\right\|}^{2}}{2\sigma_{r}^{2}}\right) \end{array}\right.\text{.} \end{array}$$

**Figure 3:**
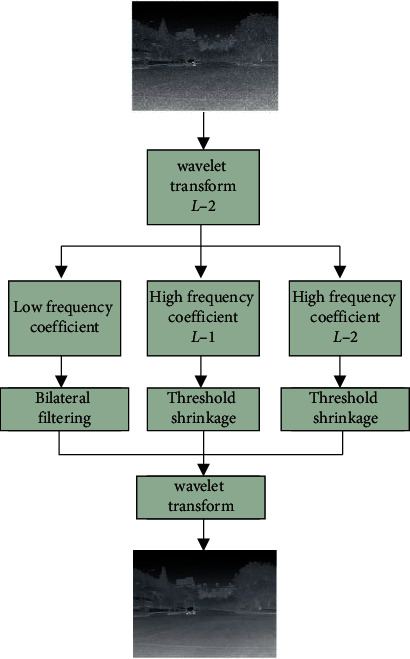
Schematic diagram of CT image filtering algorithm based on wavelet transformation and bilateral filtering.

In formula (13), *σ*_*d*_ represents the variance of the Gaussian kernel. The bilateral filter belongs to a variant of the Gaussian filter. The original image signal is the recovered signal obtained by convolution processing with the Gaussian kernel. If the signal is closer to the pixel space, the weighted value at the approximate point will be higher, so as to contribute better to the image filtering processing and retain the edge details. Combining the advantages of the wavelet threshold and the bilateral filtering, denoising the image will obtain a better processing effect of image edge detail [20]. As shown in Figure 3, the principle diagram of CT image filtering algorithm [21–25] of wavelet transformation and bilateral filtering is shown.

## 3. Experimental Test of Filtering Algorithm

**Figure 4:**
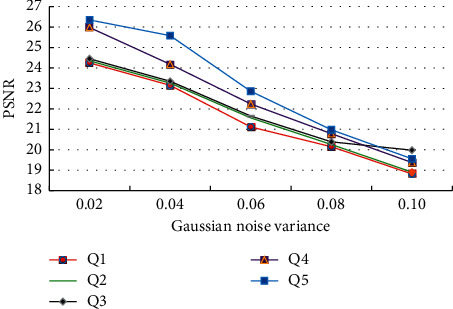
Test results of peak signal-to-noise ratio of different algorithms.

In order to further verify the effect of the proposed wavelet transformation and bilateral filtering algorithm in CT image denoising [26, 27], the human visual system is used to construct the evaluation form of mathematical model. The effect of image denoising is evaluated by peak signal-to-noise ratio (PSNR), structural similarity (SSIM), and mean square error (MSE). Among them, the larger the PSNR value is, the lower the image distortion is, and the better the image quality is. SSIM is the reference image, and the evaluation map has the similarity between structures. The higher the value is, the better the image quality is. MSE is the degree of difference between the response estimator and the estimated value. The clearer the value is, the smaller the image error is, and the better the quality is. The Shepp Logon body membrane data set was used to test oral CT images. In the experiment, the mean value of Gaussian noise was set to 0, and the variance of Gaussian noise was set to 0.04. Various CT image filtering methods were used to verify the image filtering effect of the proposed algorithm, including wavelet threshold denoising (Q1), bilateral filtering combined denoising (Q2), the dual-tree complex wavelet transformation (Q3), and nonlocal mean filter denoising (Q4), which are compared with the experimental data of the proposed wavelet transformation and bilateral filter denoising (Q5). As shown in Figure 4, the image processing results of multi-filtering algorithm under different Gaussian noise variances are shown.

It shows the image denoising results of different image filtering algorithms in Figure 4, in which the proposed wavelet transformation and bilateral filtering denoising algorithms have the highest PSNR scores. When the variances of Gaussian noise are 0.02, 004, 0.06, 0.08, and 0.10, the PSNR values of wavelet transformation and bilateral filtering algorithm are 26.35, 25.58, 22.86, 20.98, and 19.56, respectively. It can be seen that wavelet transformation and bilateral filtering algorithm can obtain the best PSNR value in different image noises and retain the best image quality. At the same time, it can be seen from the graphic data that with the expansion of the variance of Gaussian noise, the increase of image noise makes the denoising performance of each image decline, but the proposed filtering algorithm has the best performance.

**Figure 5:**
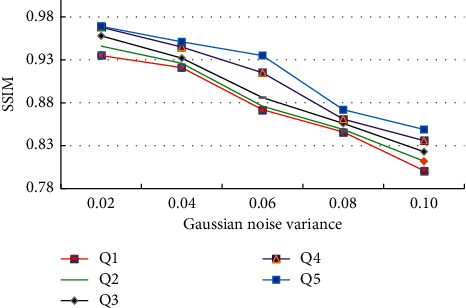
Test results of structural similarity of different algorithms.

At the same time, the SSIM performance results of each algorithm are tested in different Gaussian noise variances [28–36] as shown in Figure 5.

It shows the SSIM performance test results of multiple algorithms in Figure 5. From the visual point of view, the proposed wavelet transformation and bilateral filtering algorithm still have excellent performance. When the Gaussian noise variances are 0.02, 004, 0.06, 0.08, and 0.10, the SSIM values of wavelet transformation and bilateral filtering algorithm are 0.935, 0.946, 0.958, 0.968, and 0.969, respectively. The performance is still the best, and the best image quality can be obtained. At the same time, with the expansion of Gaussian noise variance and the increase of test image noise, the SSIM value of each algorithm has decreased, but the wavelet transformation and bilateral filtering algorithm still achieve the best performance.

**Figure 6:**
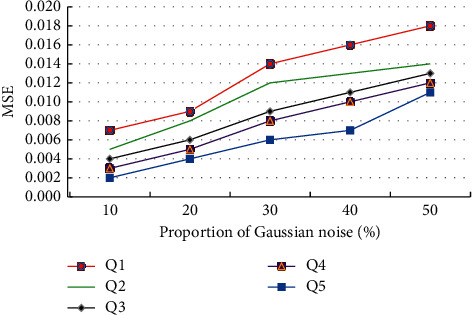
Mean square error test results of different algorithms.

In order to improve the performance of the proposed algorithm in image denoising, the MSE values of each algorithm will be tested in Gaussian noise as shown in Figure 6.

It shows the MSE results of multiple algorithms in different Gaussian noises in Figure 6. With the increase of Gaussian noise in the image, the MSE error results of each algorithm increase. Among them, the best performance of error performance is wavelet transformation and bilateral filtering algorithm, followed by nonlocal mean filtering denoising algorithm. When the proportions of Gaussian noise are 10%, 20%, 30%, 40%, and 50%, the MSE error values are 0.002, 0.004, 0.006, and 0.007, respectively. The error performance of the algorithm in image denoising is excellent.

**Figure 7:**
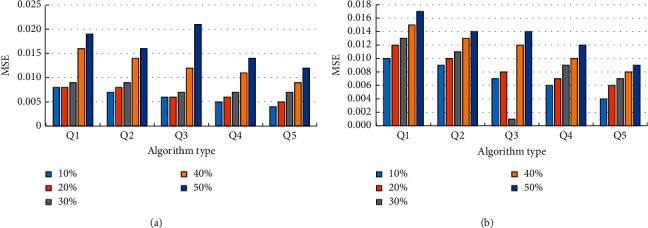
Mse error performance of different algorithms in different noise types. (a) Salt and pepper noise and (b) speckle noise.

The mean square error performance of different algorithms in speckle noise and salt and pepper noise is tested, and the results are shown in Figure 7.

It shows the MSE error performance of different algorithms in different noise types in Figure 7. In Figure 7(a), it is the mean square error results of multiple algorithms in salt and pepper noise. It can be seen that the MES error performance of wavelet transformation and bilateral filtering algorithm is the best. When the proportions of salt and pepper noise are 10%, 20%, 30%, 40%, and 50%, the MES errors are 0.004, 0.005, 0.007, 0.009, and 0.012, respectively, and the MES error performance is the best. In Figure 7(b), the result of speckle noise error is shown. The error performance of the proposed algorithm is still the best in speckle noise. When the proportions of speckle noise are 10%, 20%, 30%, 40%, and 50%, the MES errors are 0.004, 0.005, 0.007, 0.009, and 0.012, respectively, and the MSEs are 0.004, 0.006, 0.007, 0.008, and 0.009, respectively.

**Figure 8:**
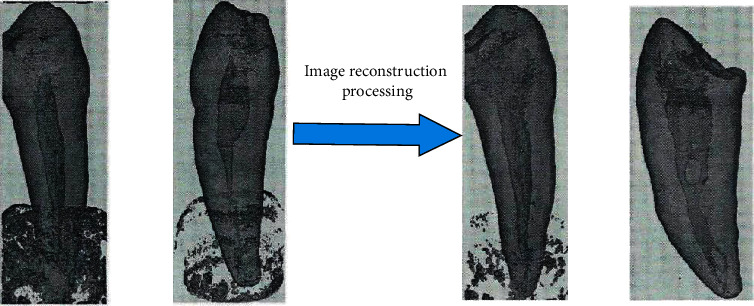
The reconstruction of isolated teeth. (a) Original reconstruction of tooth No.1, (b) Original reconstruction of tooth No.2, (c) Reconstruction of tooth No.1 after adjustment, (d) Adjustment and reconstruction of tooth No.2.

Finally, through the proposed algorithm, the teeth CT image is filtered to realize the reconstruction of the three-dimensional teeth image, and the root canal shape and volume change are evaluated to provide the main reference for oral medical treatment. It shows the reconstructed three-dimensional structure diagram of teeth in Figure 8.

It can be seen from the reconstruction data chart in Figure 8 that there are differences in the three-dimensional reconstruction results of different teeth parts. In the original CT image, there are many impurities in the compressed root after the reconstruction of Figures 8(a) and 8(b), which affect the appearance. In order to reduce the visual interference in the pressure root area, the proposed algorithm is used to denoise the image, and Figures 8(c) and 8(d) are obtained. It can be clearly seen that most of the interference in the roots of the teeth has been removed, and the condition of the teeth can be clearly judged. It can be seen that the proposed wavelet transform and bilateral filtering algorithm have excellent performance and meet the requirements of dental CT image denoising.

## 4. Conclusion

In the field of modern stomatology, the problem of oral CT image noise will affect the medical work when diagnosing the condition of patients. An image filtering algorithm for oral CT is proposed, which combines wavelet and bilateral filtering to filter the image. The low-frequency wavelet coefficients are processed by bilateral filtering, and the high-frequency coefficients are processed by local adaptive threshold filtering. The test results show that when the variances of Gaussian noise are 0.02, 004, 0.06, 0.08, and 0.10, the PSNR values of the proposed wavelet transformation and bilateral filtering algorithm are 26.35, 25.58, 22.86, 20.98, and 19.56, respectively, which have the best performance in multiple algorithms. At the same time, the proposed algorithm has the best performance in CT image denoising in structural similarity (SSIM) and mean square error (MSE). It can be seen that wavelet transformation and bilateral filtering algorithm can effectively denoise oral CT images and meet the requirements of medical workers. However, there are also deficiencies in the research content. It is only for the oral CT image processing scene. In the future, we should consider applying it to more images to improve the denoising performance of the algorithm.

## Data Availability

The dataset can be accessed upon request.
